# The Prognostic Capacity of the Radiographic Assessment for Lung Edema Score in Patients With COVID-19 Acute Respiratory Distress Syndrome—An International Multicenter Observational Study

**DOI:** 10.3389/fmed.2021.772056

**Published:** 2022-01-05

**Authors:** Christel M. A. Valk, Claudio Zimatore, Guido Mazzinari, Charalampos Pierrakos, Chaisith Sivakorn, Jutamas Dechsanga, Salvatore Grasso, Ludo Beenen, Lieuwe D. J. Bos, Frederique Paulus, Marcus J. Schultz, Luigi Pisani

**Affiliations:** ^1^Department of Intensive Care and Laboratory of Experimental Intensive Care and Anesthesiology (L·E·I·C·A), Amsterdam UMC, Amsterdam, Netherlands; ^2^Department of Emergency and Organ Transplantation, University of Bari Aldo Moro, Bari, Italy; ^3^Department of Anaesthesiology and Critical Care, Hospital Universitario y Politecnico la Fe, Valencia, Spain; ^4^Perioperative Medicine Research Group, Instituto de Investigación Sanitaria la Fe, Valencia, Spain; ^5^Department of Intensive Care, Centre Hospitalier Universitaire Brussels, Brussels, Belgium; ^6^Department of Clinical Tropical Medicine, Mahidol University, Bangkok, Thailand; ^7^Division of Pulmonary and Critical Care, Department of Medicine, Chonburi Hospital, Chonburi, Thailand; ^8^Department of Radiology, Amsterdam UMC, Amsterdam, Netherlands; ^9^Department of Pulmonology, Amsterdam UMC, Amsterdam, Netherlands; ^10^Center of Expertise Urban Vitality, Faculty of Health, Amsterdam University of Applied Sciences, Amsterdam, Netherlands; ^11^Mahidol-Oxford Tropical Medicine Research Unit (MORU), Mahidol University, Bangkok, Thailand; ^12^Nuffield Department of Medicine, University of Oxford, Oxford, United Kingdom; ^13^Anaesthesia and Intensive Care Unit, Miulli Regional Hospital, Acquaviva delle Fonti, Italy

**Keywords:** intensive and critical care, ARDS, corona virus (COVID-19), mechanical ventilated, chest X-ray (CXR), RALE score, prognostication, radiograph (X-ray)

## Abstract

**Background:** The radiographic assessment for lung edema (RALE) score has an association with mortality in patients with acute respiratory distress syndrome (ARDS). It is uncertain whether the RALE scores at the start of invasive ventilation or changes thereof in the next days have prognostic capacities in patients with COVID-19 ARDS.

**Aims and Objectives:** To determine the prognostic capacity of the RALE score for mortality and duration of invasive ventilation in patients with COVID-19 ARDS.

**Methods:** An international multicenter observational study included consecutive patients from 6 ICUs. Trained observers scored the first available chest X-ray (CXR) obtained within 48 h after the start of invasive ventilation (“baseline CXR”) and each CXRs thereafter up to day 14 (“follow-up CXR”). The primary endpoint was mortality at day 90. The secondary endpoint was the number of days free from the ventilator and alive at day 28 (VFD-28).

**Results:** A total of 350 CXRs were scored in 139 patients with COVID-19 ARDS. The RALE score of the baseline CXR was high and was not different between survivors and non-survivors (33 [24–38] vs. 30 [25–38], *P* = 0.602). The RALE score of the baseline CXR had no association with mortality (hazard ratio [HR], 1.24 [95% CI 0.88–1.76]; *P* = 0.222; area under the receiver operating characteristic curve (AUROC) 0.50 [0.40–0.60]). A change in the RALE score over the first 14 days of invasive ventilation, however, had an independent association with mortality (HR, 1.03 [95% CI 1.01–1.05]; *P* < 0.001). When the event of death was considered, there was no significant association between the RALE score of the baseline CXR and the probability of being liberated from the ventilator (HR 1.02 [95% CI 0.99–1.04]; *P* = 0.08).

**Conclusion:** In this cohort of patients with COVID-19 ARDS, with high RALE scores of the baseline CXR, the RALE score of the baseline CXR had no prognostic capacity, but an increase in the RALE score in the next days had an association with higher mortality.

## Introduction

Patients with coronavirus disease 2019 (COVID-19) frequently develop acute respiratory distress syndrome (ARDS), mandating intensive care unit (ICU) admission, usually for invasive ventilation (1, 2). Outcome prediction in these patients could use the classification based on the severity of oxygenation problems (3), albeit that this approach has been shown to be not so successful, at least not in patients with ARDS due to another cause (4). The chest radiograph (CXR) is a routine imaging tool for critically ill patients that receive invasive ventilation (5, 6) and could contribute to defining severity, progression, and complications and maybe also predict outcomes from COVID-19 (7, 8). One important drawback of the CXR, however, is the poor interobserver reliability in qualitative visual scoring of pulmonary opacifications (9).

The radiographic assessment for lung edema (RALE) score is a numeric scoring system, recently introduced in an attempt to improve the quantification of pulmonary abnormalities on the CXR. For this score, each quadrant of the chest at the CXR is scored for the extent of consolidations and density of opacities to define the extent and severity of lung parenchymal abnormalities. The RALE score has not only been found to have excellent diagnostic accuracy (10–12) but also to have the prognostic capacity in patients with ARDS due to COVID-19 (13, 14).

We hypothesized that the RALE score has the prognostic capacity in patients with COVID-19 ARDS (15, 16). In this international study, we determined the prognostic capacity of the RALE score of the first available CXRs that was obtained under invasive ventilation for COVID-19 ARDS. We also wished to determine the prognostic capacity of changes in the RALE score over the first 14 days after initiation of invasive ventilation.

## Methods

### Study Design

This is an international, multicenter, retrospective observational study in invasively ventilated patients with COVID-19 admitted to participating ICUs between December 1, 2019, and May 31, 2020. The study enrolled ICU patients in the Amsterdam UMC, location AMC, Amsterdam, The Netherlands; University of Bari Policlinic Hospital, Bari, Italy; Miulli Regional Hospital, Acquaviva Delle Fonti, Italy; Centre Hospitalier Universitaire Brussels, Brussels, Belgium; Mahidol University Hospital in Bangkok, Thailand; and Chonburi Hospital, Chonburi, Thailand. The study protocol was initially approved by the institutional review board of the Amsterdam UMC, location AMC, Amsterdam, The Netherlands (approval letter W20_311 # 20.346). Thereafter, the protocol was approved in other hospitals. The need for individual patient informed consent was waived because of the observational nature of the study. The study is registered at clinicaltrials.gov (trial identification number NCT 04485338).

### Inclusion and Exclusion Criteria

Consecutive patients were included if (1) admitted to one of the participating ICUs, (2) received invasive ventilation; and (3) with ARDS due to COVID-19 that was confirmed by reverse transcriptase-PCR. Patients were excluded if aged <18 years of age, when COVID-19 was not the reason for invasive ventilation, or if there was no CXR within 48 h after starting intubation.

### Data Collection

An online case report form (www.castoredc.com) was used to collect and store the study data. Baseline and demographic characteristics included age, gender, body mass index (BMI); severity indexes, such as the acute physiology and chronic health evaluation (APACHE) II and IV score and the Sequential Organ Failure Assessment (SOFA); and ventilation characteristics at the moment of the CXR, such as FiO_2_, positive end-expiratory pressure (PEEP), maximum airway pressure (Pmax), respiratory rate, tidal volume, and the nearest blood gas analysis results.

We collected all CXRs that were taken within the first 14 days after the start of invasive ventilation from each electronic imaging system and uploaded de-identified CXRs in JPEG format into the database.

### RALE Scoring

The RALE score was calculated as described before (11, 13). In short, the chest at the CXR was divided into 4 quadrants by a vertical line over the spine and a horizontal line at the level of the first branch of the left main bronchus; each quadrant was then scored for the extent of alveolar opacities (*consolidation* score, from 0 to 4) and the corresponding density of alveolar opacities (*density* score, from 1 to 3) (Supplementary Figure 1). In case no consolidations were visible, the consolidation score was “0,” and density was not scored. The final score is the sum of the product of the consolidation and density scores for each quadrant. The RALE score ranges from 0 (no abnormalities) to 48 (maximum abnormalities), where in a recent study, patients with ARDS have RALE scores that range from 15 to as high as 26 (16). Among patients with ARDS, the baseline RALE score is not associated with the ARDS severity groups by P/F ratio (16).

Every CXR was scored by at least two independent scorers that were extensively educated in calculating RALE scores. For this, each scorer was trained in the RALE score by one of the investigators (CZ), who was trained during a 1-month focused period by the team that developed the RALE score (11). An interclass correlation coefficient (ICC) > 0.8 between the trainer and other scorers on a training sample of 22 CXRs from another set of CXRs of patients with ARDS was a prerequisite for scoring CXRs in the study dataset. A third scorer was involved only if the difference in numeric RALE score between two scorers was >25%, to reach a final consensus.

### Endpoints

The primary endpoint was 90-day mortality; the secondary endpoint was the number of days free from the ventilator and alive at day 28 (VFD-28).

The ventilator and alive at day 28 was calculated as the number of days that a patient was alive and free of invasive ventilation if the period of unassisted breathing lasted > 24 consecutive hours. Patients who died or received invasive ventilation for more than 28 days had the lowest number of VFD-28, i.e., 0 days.

### Statistical Analysis

We did not perform a formal sample size calculation—instead, the available patients served as the sample size for this study.

Demographic data and outcomes are summarized as mean (SD) or medians (interquartile range) for continuous variables and as frequencies (percentage) for categorical variables. In the case of normally distributed, continuous variables were compared between groups with a *t*-test or ANOVA. When not considered normally distributed, continuous variables were compared between groups with Mann–Whitney U test or Kruskal–Wallis test, as appropriate. Categorical variables were compared between groups by chi-square analysis. Missing data imputations were performed by random forest whenever any variable included in the analysis showed a missing data percentage of >10%.

Interobserver variability was assessed using ICC with a two-way mixed agreement model. Bland–Altman plots were used to visualize the aggregate agreement between the two scorers initially assessing a CXR.

The first CXR was labeled “baseline CXR.” The association between the RALE score of the baseline CXR with mortality as a time-to-event was analyzed with a Cox regression model, reporting the hazard ratio (HR) with a 95% CI. Herein, baseline RALE score was used as a continuous numerical variable, while age, gender, pH, and lactate were entered as covariates. In an additional Cox model, the RALE score was categorized into quartiles. Herein, proportionality assumptions were checked by Schoenfeld and martingale residuals and influential observations. The predictive accuracy of the RALE score of the baseline CXR for 90-day mortality was also described by the area under the receiver operating characteristics curve (AUROC) with 95% CI.

The association of the baseline RALE score with VFDs was tested using a competing risk model with extubation and death as the events of interest. The results are described with the use of cumulative incidence function and reported as sub-distribution HR with 95% CI estimated from a Fine–Gray model (12).

To assess the association of changes in the RALE score overtime in the first 14 days from onset of mechanical ventilation of consecutive CXRs with mortality, we use a joint model fitting repeated RALE scores with a mixed model and mortality as a time-to-event variable using the same covariates specified in the previous models. The joint model combines Cox regression and linear mixed-effects (LME) models, where the LME part of the models estimates the linear change pattern of the RALE score over follow-up time.

All analyses were performed using a two-sided superiority hypothesis test, with a significance level of 0.05, and presented with a two-sided 95% CI. No corrections were performed for multiple comparisons across secondary clinical outcomes, thus, these findings should be considered exploratory. Analyses were performed using software R (version 4.0.2, R Core Team, 2016, Vienna, Austria).

## Results

### Patients

From December 1, 2019, to May 31, 2020, 178 patients were screened in 6 ICUs. We excluded 36 patients from the analysis because a baseline CXR was missing and 3 other patients because of incomplete data (Supplementary Figure 2). In the remaining 139 patients, 350 CXRs were available within the first 14 days of invasive ventilation.

Baseline characteristics, ventilation characteristics, and outcomes are presented in Tables 1, 2. The median age was 65 [59–74] years; the most common comorbidities were hypertension and diabetes. The majority of patients had moderate-to-severe ARDS with low lung compliance. Patients who did not survive had no improvement in lung compliance nor the RALE score compared to survivors during the first 14 days (Supplementary Figure 3). Non-survivors did receive a higher FiO_2_, PEEP, and peak pressure than survivors. The crude 90-day mortality was high, 61.2%.

**Table 1:** Baseline characteristics of the patients.

	**Overall (*n* = 139)**	Alive^*^ (*n* = 54)	**Dead (*n* = 85)**	***P*-value**	**SMD**
**Demographics**					
Age, years (median [IQR])	65 [59–74]	61.0 [55–71]	69 [60–75]	0.002	0.505
Male gender—no (%)	65 (46.8)	27 (50.0)	38 (44.7)	0.663	0.106
Body mass index, kg·m^2^ (median [IQR])	27.3 [24.7–30.0]	26.9 [24.2–29.4]	27.5 [25.4–30.0]	0.595	0.086
**Comorbidities and severity**					
**Co-existing disorders—no (%)**					
Hypertension—no (%)	70 (50.4)	25 (46.3)	45 (52.9)	0.555	0.133
Diabetes—no (%)	41 (29.5)	13 (24.1)	28 (32.9)	0.354	0.197
Chronic obstructive pulmonary disease—no (%)	23 (16.5)	7 (13.0)	16 (18.8)	0.502	0.161
Cardiovascular disease—no (%)	17 (12.2)	3 (5.6)	14 (16.5)	0.099	0.354
None—no (%)	17 (12.2)	9 (16.7)	8 (9.4)	0.314	0.217
Other—no (%)	60 (43.2)	14 (25.9)	46 (54.1)	0.002	0.601
APACHE II (median [IQR])	15 [12–20]	15 [12–18]	15 [12–20]	0.132	0.318
SOFA score (median [IQR])	6 [4–8]	6 [4–8]	5 [4–8]	0.481	0.033
**Outcomes**					
Survival time, days (median [IQR])	26 [19–90]	90 [90–90]	11 [7–21]	<0.001	4.525
ICU length of stay, days (median [IQR])	12 [70–20]	12 [6–21]	11 [7–20]	0.416	0.285
Hospital length of stay, days (median [IQR])	15 [8–29]	26 [15 −38]	11 [7–21]	<0.001	0.214

*Data are median (quartile 25%–quartile 75%), mean (±SD) or No (%). Percentages may not total 100 because of rounding*.

*ICU, intensive care unit; APACHE, acute physiology and chronic health evaluation; SOFA, sequential organ failure assessment*.

* *At day 90*.

**Table 2:** Ventilation parameters measured with the baseline chest radiography.

	**Overall (*n* = 139)**	Alive^*^ (*n* = 54)	**Dead (*n* = 85)**	***P*-value**	**SMD**
**Ventilatory parameters**					
**Ventilation mode—*****n*** **(%)**				0.252	0.413
Pressure controlled	36 (28.1%)	11 (21.6%)	25 (32.5%)		
Pressure support	7 (5.5%)	5 (9.8%)	2 (2.6%)		
Volume controlled	53 (41.4%)	20 (39.2%)	33 (42.9%)		
ASV/Intellivent	8 (6.2)	3 (5.9%)	5 (6.5%)		
Spontaneous	24 (18.8)	12 (23.5%)	12 (15.6%)		
FiO_2_, % (median [IQR])	70 [50–90]	55 [40–80]	80 [60–100]	0.001	0.663
PaO_2_/FiO_2_ (median [IQR])	130 [88–175]	138 [96–184]	112 [86–152]	0.082	0.294
Tidal volume set, ml (median [IQR])	450 [410–500]	475 [403–500]	450 [415–495]	0.532	0.236
Tidal volume measured, ml (median [IQR])	450 [400–500]	458 [378–512]	450 [408–500]	0.852	0.069
Tidal volume, ml/kg PBW (median [IQR])	7.0 [6.3–7.5]	6.6 [6.0–7.6]	7.2 [6.5–7.6]	0.065	0.298
Respiratory rate, breaths/min (median [IQR])	20 [17–26]	24 [18–26]	20 [16–25]	0.134	0.225
Peak pressure, cmH_2_O (median [IQR])	25 [18–29]	23 [17–26]	26 [21–30]	0.017	0.464
PEEP, cmH_2_O (median [IQR])	10 [8–12]	9 [8–10]	10 [8–12]	0.013	0.378
Plateau pressure, cmH_2_O (median [IQR])	25 [23–28]	25 [21–26]	26 [24–30]	0.281	0.578
Dynamic compliance, ml/cmH_2_O (median [IQR])	29 [21–51]	34 [23–47]	26 [20–61]	0.491	0.113

*Data are median (quartile 25%–quartile 75%), mean (±SD) or No (%). Percentages may not total 100 because of rounding*.

*PEEP, positive end-expiratory pressure; ASV, adaptive support ventilation; FiO_2_, fraction of inspired oxygen; PaO_2_, partial pressure of oxygen*.

* *At day 90*.

### RALE Scoring

**Figure 1:**
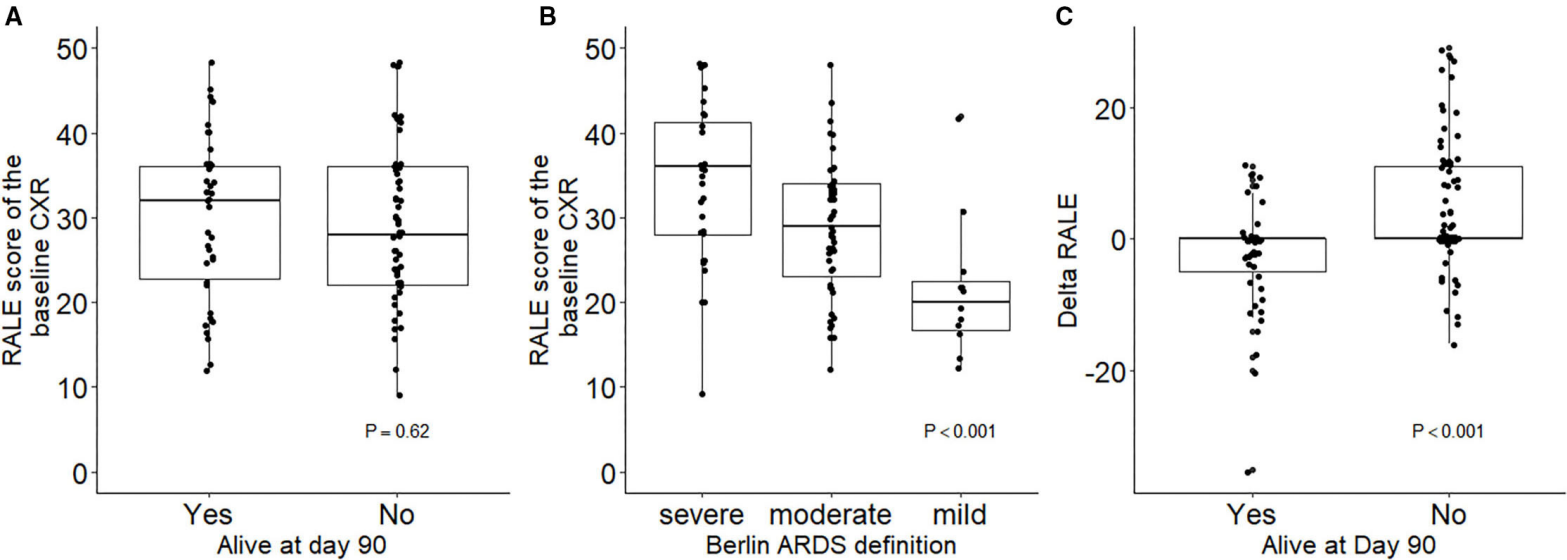
Baseline RALE scores in survivors vs. non-survivors **(A)** and patients with different ARDS severity **(B)**. Changes in RALE score across the first 14 days after onset of invasive ventilation in survivors vs. non-survivors **(C)**. RALE, radiographic assessment for lung edema; ARDS, acute respiratory distress syndrome.

The interobserver agreement was high (ICC, 0.95 [95% CI 0.93–0.96]). In 14 of 350 (4%) CXRs, a third scorer was needed to reach the final consensus. Bland–Altman plots are shown in Supplementary Figure 4. The RALE scores of baseline CXRs were high, with a median RALE score of 32 [24–38], but comparable between survivors and non-survivors (Figure 1A). The RALE score of the baseline CXR was increased with worsening of ARDS severity (Figure 1B). Linear regression analysis showed how the RALE had no significant association with the dynamic compliance, with an R^2^ of 0.0001.

### The Prognostic Capacity of the RALE Score of the Baseline CXR

**Figure 2:**
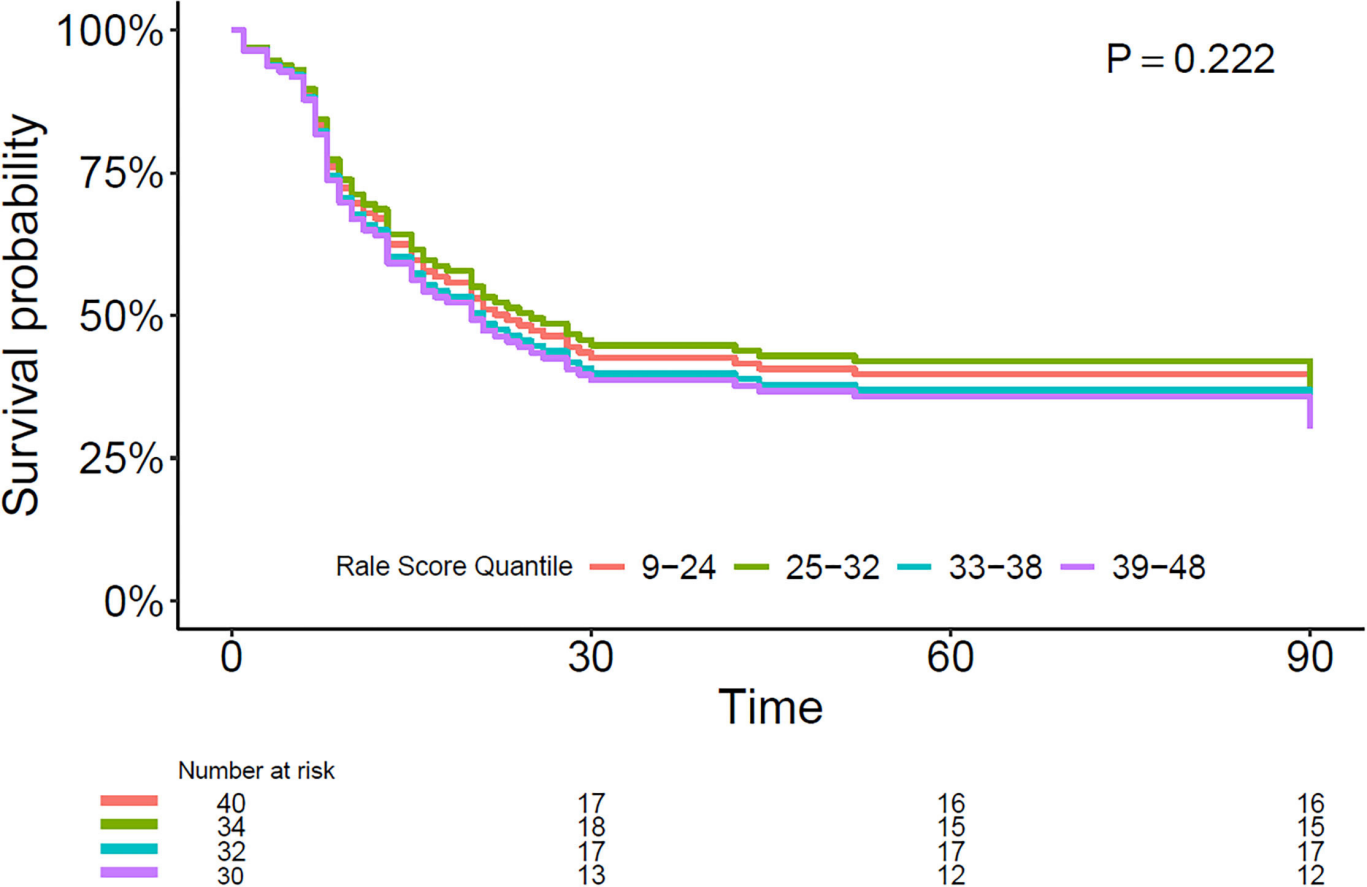
Multivariable Cox model with survival estimates during the 90-day follow-up period in patients stratified by baseline RALE score quartiles. RALE, radiographic assessment for lung edema.

**Figure 3:**
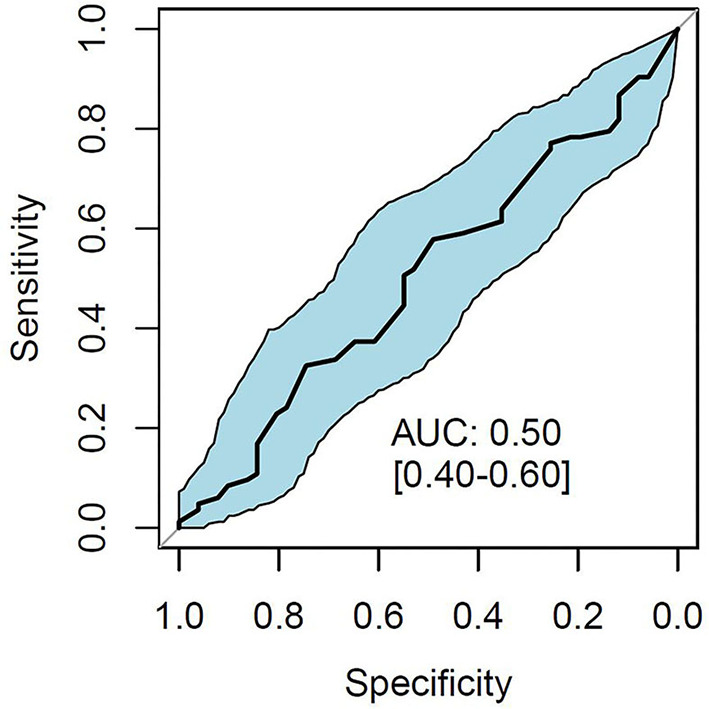
Discriminative capacity of the baseline RALE score for 90-day mortality. AUC, area under the curve; RALE, radiographic assessment for lung edema.

The RALE score of the baseline CXR had no association with mortality (HR, 1.24 [95% CI 0.88–1.76]; *P* = 0.222). Estimates of 90 days survival in patients stratified by quartiles of the baseline RALE score is shown in Figure 2. There was no difference in survival between the quartiles after adjusting for age, gender, arterial pH, and plasma lactate. The baseline RALE score had no prognostic capacity for mortality (Figure 3).

### The Prognostic Capacity of Changes in RALE Score

The change in the RALE score over time was different between survivors and non-survivors (Figure 1C). An increase in the RALE score until day 14 had an independent association with mortality (HR, 1.03 [95% CI 1.01–1.05]; *P* < 0.001). In other words, for every point increase in the RALE score over time the risk of death increased by 3% [95% CI 1–5%]. Supplementary Figure 5 shows how the gradual worsening in RALE score over time increases the probability of death at 28 days.

### Association Between the RALE Score and VFD-28

Overall patients had 0 [0–14] VFD-28, where survivors had 18 [11–23] VFD-28 and a duration of invasive mechanical ventilation of 10 [6–16] days. When the event of death was considered, there was no significant association between the RALE score of the baseline CXR and the probability of being liberated from the ventilator (HR 1.02 [95% CI 0.99–1.04]; *P* = 0.08).

## Discussion

The findings of this international multicenter study in patients with COVID-19 ARDS can be summarized as follows: (i) the extent and severity of parenchymal damage quantified by the RALE score were very high in survivors and non-survivors; (ii) the RALE score of the baseline CXR was neither associated with mortality in the first 90 days nor with successful liberation from invasive ventilation; and (iii) a worsening of the RALE score over the first 14 days of invasive ventilation was associated with an increased risk of death.

This study has several strengths. The study was designed to minimize bias by strictly adhering to a predefined statistical analysis plan and training of scorers. There was a minimal loss to follow-up. We had a low interobserver variability between the scorers, confirming the feasibility and reliability of the RALE score (11, 12, 16, 17). Finally, patients were enrolled in 6 hospitals in 4 different countries and included patients in university hospitals, teaching and non-teaching hospitals, contributing to the generalizability of the findings.

Counter to our hypothesis, we did not find an association of the RALE score of the baseline CXR with mortality or liberation from mechanical ventilation. We could even not find an association with mortality in the quartile with the highest RALE scores. The findings of our study are in line with those from a recent study that could not establish an association between RALE and mortality in ICU patients with COVID-19 (18). Contrasting, the baseline RALE score in patients with COVID-19 presenting to the emergency department did predict adverse outcomes (19) and also in patients with less severe COVID-19 ARDS located outside of an ICU (15). However, another study with a lower median RALE score in both survivors and non-survivors confirmed the capacity of the RALE score to predict adverse outcomes, defined as death or need for invasive ventilation (10). Our findings ultimately suggest that, when baseline RALE scores are high, the RALE score may not be helpful in predicting mortality and the chance of liberation from the ventilator.

Studies that assessed the prognostic capacity of the RALE score in patients with ARDS due to another cause than COVID-19 had conflicting results. The baseline RALE score did not predict outcome in these patients (16). Another study did find an association of baseline RALE with mortality (14). However, patients in this latter study showed very similar baseline RALE between survivors and non-survivors, just as in our cohort. In another RALE study, there was an association between the baseline RALE score and 28-day mortality, but without an association with VFDs (13). The median RALE score in our cohort of patients with COVID-19 ARDS was much higher than the RALE score reported in patients with ARDS due to another cause (12, 14–16, 20). For instance—in patients with ARDS included in the original study that reported on the RALE score, the RALE score was 27 [18–35] (13). Moreover, patients with a RALE score >30, frequently seen in our cohort, were in the highest quartile in a recent secondary analysis of another non-COVID ARDS trial (14). Whether COVID-19 ARDS is characterized by important pathophysiological differences compared to classical ARDS is still debated (21–24). In our cohort, median values of compliance were consistently low and in line with findings in previous studies (25, 26). Furthermore, our study confirmed the association between the change in RALE score over time and outcome, already identified in a recent study conducted on ARDS patients due to other etiologies (14). The change of RALE score over time was independently associated with outcome. This confirms recent findings in patients with COVID-19 in which the RALE score predicted mortality and the need for invasive ventilation (10). Furthermore, an increase in the RALE score was found to be associated with a prolonged need for invasive ventilation and with a lower number of VFDs in ICU patients with ARDS (16). In addition, the prognostic effect of early changes in RALE score in moderate-to-severe ARDS has been confirmed by another study in 135 ICU patients (14). The consistency of these findings, added to the feasibility of repeated bedside CXRs in patients with COVID-19, allows for the changes in RALE score to be used as an increasingly established prediction tool.

The RALE score is an easy reproducible tool that can be easily computed after a CXR is made. The interobserver variability we found is comparable to one of the original studies (11) and subsequent investigations (12–14, 16). Although dedicated CXR apparatuses are still not ubiquitous in ICUs in some low- and middle-income countries (27), it is considered a routine imaging tool for patients who receive invasive ventilation (5, 6). Conversely, CT is a more costly and less available imaging technique, with feasibility issues in patients with COVID-19 and with a radiation load that is much higher than that of a CXR (28). The findings of this and previous studies suggest that the RALE score is an attractive visual metric, especially in settings with low resources.

This study has several limitations. The retrospective design limits the inclusion of all potential confounders. The sample size of this study was relatively small. However, the narrow CI suggests that repeating the study on a larger sample is unlikely to change the result of a significant association between change in RALE score and outcome. Similarly, due to the retrospective collection of study CXRs, time points for CXRs could not be strictly predefined. The third scorer was not blinded for the previous scores, and this could have generated scoring bias. However, high interobserver variability between first and second scorers was only found in <5% of CXRs.

## Conclusions

In this cohort of patients with COVID-19 ARDS, the RALE score of the baseline CXR was neither associated with 90-day mortality nor with the probability of being liberated from the ventilator. However, an increase in the RALE score over the next days had an association with higher mortality.

## Data Availability Statement

The raw data supporting the conclusions of this article will be made available by the authors on motivated request.

## Ethics Statement

The studies involving human participants were reviewed and approved by Medical Ethical Board AMC. Written informed consent for participation was not required for this study in accordance with the national legislation and the institutional requirements.

## Author Contributions

All authors listed have made a substantial, direct, and intellectual contribution to the work and approved it for publication.

## Funding

This work was supported by the Department of Intensive Care, Amsterdam UMC, Location AMC, Amsterdam.

## Conflict of Interest

The authors declare that the research was conducted in the absence of any commercial or financial relationships that could be construed as a potential conflict of interest.

## Publisher's Note

All claims expressed in this article are solely those of the authors and do not necessarily represent those of their affiliated organizations, or those of the publisher, the editors and the reviewers. Any product that may be evaluated in this article, or claim that may be made by its manufacturer, is not guaranteed or endorsed by the publisher.

## References

[1] Grasselli G Zangrillo A Zanella A Antonelli M Cabrini L Castelli A. Baseline Characteristics and Outcomes of 1591. patients infected with SARS-CoV-2 admitted to ICUs of the Lombardy Region, Italy. JAMA. (2020) 20:5394. 10.1001/jama.2020.5394 32250385 PMC7136855

[2] Guan WJ Ni ZY Hu Y Liang WH Ou CQ He JX. Clinical characteristics of coronavirus disease 2019 in China. N Engl J Med. (2020) 9:1404–12. 10.1056/NEJMoa2002032 32109013 PMC7092819

[3] Ranieri VM Rubenfeld GD Thompson BT Ferguson ND Caldwell E Fan E. Acute respiratory distress syndrome: the Berlin Definition. JAMA. (2012) 307:2526–33. 10.1001/jama.2012.5669 22797452

[4] Ferguson ND Fan E Camporota L Antonelli M Anzueto A Beale R. The Berlin definition of ARDS: an expanded rationale, justification, and supplementary material. Intensive Care Med. (2012) 38:1573–82. 10.1007/s00134-012-2682-1 22926653

[5] Trotman-Dickenson B. Radiology in the intensive care unit (Part I). J Intens Care Med. (2003) 18:198–210. 10.1177/0885066603251897 15035766

[6] Graat ME Hendrikse KA Spronk PE Korevaar JC Stoker J Schultz MJ. Chest radiography practice in critically ill patients: a postal survey in the Netherlands. BMC Med Imaging. (2006) 6:8. 10.1186/1471-2342-6-8 16848892 PMC1557847

[7] Lomoro P Verde F Zerboni F Simonetti I Borghi C Fachinetti C. COVID-19 pneumonia manifestations at the admission on chest ultrasound, radiographs, and CT: single-center study and comprehensive radiologic literature review. Eur J Radiol Open. (2020) 7:100231. 10.1016/j.ejro.2020.100231 32289051 PMC7129441

[8] Prokop M van Everdingen W van Rees Vellinga T Quarles van Ufford H Stoger L Beenen L. CO-RADS: A Categorical CT assessment scheme for patients suspected of having Covid-19-definition and evaluation. Radiology. (2020) 296:E97–E104. 10.1148/radiol.2020201473 32339082 PMC7233402

[9] Rubenfeld GD Caldwell E Granton J Hudson LD Matthay MA. Interobserver variability in applying a radiographic definition for ARDS. Chest. (1999) 116:1347–53. 10.1378/chest.116.5.1347 10559098

[10] Ebrahimian S Homayounieh F Rockenbach M Putha P Raj T Dayan I. Artificial intelligence matches subjective severity assessment of pneumonia for prediction of patient outcome and need for mechanical ventilation: a cohort study. Sci Rep. (2021) 11:858. 10.1038/s41598-020-79470-0 33441578 PMC7807029

[11] Zimatore C Pisani L Lippolis V Calfee CS Ware LB Algera AG. The radiographic assessment of lung edema (RALE) score has excellent diagnostic accuracy for ARDS. Euro Respirat J. (2019) 54:OA3299. 10.1183/13993003.congress-2019.OA329934122143

[12] Zimatore C Pisani L Lippolis V Warren MA Calfee CS Ware LB. Accuracy of the radiographic assessment of lung edema score for the diagnosis of ARDS. Front Physiol. (2021) 12:731. 10.3389/fphys.2021.672823 34122143 PMC8188799

[13] Warren MA Zhao Z Koyama T Bastarache JA Shaver CM Semler MW. Severity scoring of lung oedema on the chest radiograph is associated with clinical outcomes in ARDS. Thorax. (2018) 73:840–6. 10.1136/thoraxjnl-2017-211280 29903755 PMC6410734

[14] Jabaudon M Audard J Pereira B Jaber S Lefrant JY Blondonnet R. Early changes over time in the radiographic assessment of lung edema score are associated with survival in ARDS. Chest. (2020) 158:2394–403. 10.1016/j.chest.2020.06.070 32659235 PMC7768934

[15] Sensusiati AD Amin M Nasronudin N Rosyid AN Ramadhan NA Lathifah R. Age, neutrophil lymphocyte ratio, and radiographic assessment of the quantity of lung edema (RALE) score to predict in-hospital mortality in COVID-19 patients: a retrospective study. F1000Res. (2020) 9:1286. 10.12688/f1000research.26723.1 33537125 PMC7836085

[16] Kotok D Yang L Evankovich JW Bain W Dunlap DG Shah F. The evolution of radiographic edema in ARDS and its association with clinical outcomes: a prospective cohort study in adult patients. J Crit Care. (2020) 56:222–8. 10.1016/j.jcrc.2020.01.012 32028223 PMC7136845

[17] Cozzi D Albanesi M Cavigli E Moroni C Bindi A Luvarà S. Chest X-ray in new Coronavirus Disease (2019). (COVID-19) infection: findings and correlation with clinical outcome. Radiol Med. (2020). 125:730–7. 10.1007/s11547-020-01232-9 32519256 PMC7282464

[18] Herrmann J Adam EH Notz Q Helmer P Sonntagbauer M Ungemach-Papenberg P. COVID-19 induced acute respiratory distress syndrome—a multicenter observational study. Front Med. (2020) 7:995. 10.3389/fmed.2020.599533 33392222 PMC7775385

[19] Mushtaq J Pennella R Lavalle S Colarieti A Steidler S Martinenghi CMA. Initial chest radiographs and artificial intelligence (AI) predict clinical outcomes in COVID-19 patients: analysis of 697 Italian patients. Eur Radiol. (2021) 31:1770–9. 10.1007/s00330-020-07269-8 32945968 PMC7499014

[20] Todur P Srikant N Prakash P. Correlation of oxygenation and radiographic assessment of lung edema (RALE) score to lung ultrasound score (lus) in acute respiratory distress syndrome (ARDS) patients in the intensive care unit. Can J Respir Ther. (2021) 57:53–9. 10.29390/cjrt-2020-063 34041358 PMC8132988

[21] Narayan A Garg P Arora U Ray A Wig N. Pathophysiology of COVID-19-associated acute respiratory distress syndrome. Lancet Respir Med. (2021) 9:e3. 10.1016/S2213-2600(20)30509-9 33197386 PMC7831561

[22] Azoulay E Zafrani L Mirouse A Lengliné E Darmon M Chevret S. Clinical phenotypes of critically ill COVID-19 patients. Intens Care Med. (2020) 46:1651–2. 10.1007/s00134-020-06120-4 32468086 PMC8830032

[23] Tobin MJ. Pondering the atypicality of ARDS in COVID-19 is a distraction for the bedside doctor. Intens Care Med. (2021) 47:361–2. 10.1007/s00134-020-06340-8 33449135 PMC7809224

[24] Goligher EC Ranieri VM Slutsky AS. Is severe COVID-19 pneumonia a typical or atypical form of ARDS? and does it matter? Intens Care Med. (2021) 47:83–5. 10.1007/s00134-020-06320-y 33237346 PMC7686835

[25] Grasselli G Cattaneo E Florio G Ippolito M Zanella A Cortegiani A. Mechanical ventilation parameters in critically ill COVID-19 patients: a scoping review. Crit Care. (2021) 25:115. 10.1186/s13054-021-03536-2 33743812 PMC7980724

[26] Grasselli G Tonetti T Protti A Langer T Girardis M Bellani G. Pathophysiology of COVID-19-associated acute respiratory distress syndrome: a multicentre prospective observational study. Lancet Respirat Med. (2020) 8:1201–8. 10.1016/S2213-2600(20)30370-2 32861276 PMC7834127

[27] Pisani L Algera AG Serpa Neto A Ahsan A Beane A Chittawatanarat K. Epidemiological characteristics, ventilator management, and clinical outcome in patients receiving invasive ventilation in intensive care units from 10 asian middle-income countries (PRoVENT-iMiC): an international, multicenter, prospective study. Am J Trop Med Hyg. (2021) 104:1022–33. 10.4269/ajtmh.20-1177 33432906 PMC7941813

[28] U.S. Food & Drugs administration (FDA). Available online at: https://www.fda.gov/radiation-emitting-products/medical-x-ray-imaging/what-are-radiation-risks-ct#:~:text=The%20effective%20doses%20from%20diagnostic,survivors%20of%20the%20atomic%20bombs (accessed May 12, 2017).

