# Supplementary material for: The Prognostic Capacity of the Radiographic Assessment for Lung Edema Score in Patients With COVID-19 Acute Respiratory Distress Syndrome—An International Multicenter Observational Study
Source: Front Med (Lausanne). 2022 Jan 5;8:772056. doi: 10.3389/fmed.2021.772056 (PMC8766516; doi:10.3389/fmed.2021.772056)
Supplement: Supplementary file 1 [file Data_Sheet_1.pdf]

# The Prognostic Capacity of the RALE Score in COVID–19 ARDS Patients—an international multicenter observational study

Supplementary material

**eFigure 1. The RALE scoring sheet, showing the total score and the score for each of the four quadrant for a representative study patient.**

Consolidation[a]

| Consolidation Score | Extent of alveolar opacities |
|---|---|
| 0 | None |
| 1 | < 25 % |
| 2 | 25 – 50 % |
| 3 | 50 – 75 % |
| 4 | > 75 % |

Calculation of the RALE score for radiograph

| Score | Q1 | Q2 | Q3 | Q4 | Total |
|---|---|---|---|---|---|
| Consolidation | 2 | 1 | 3 | 4 | |
| Density | 3 | 3 | 3 | 3 | |
| Quadrant Score | 2 x 3 = 6 | 1 x 3 = 3 | 3 x 3 = 9 | 4 x 3 = 12 | 30 |

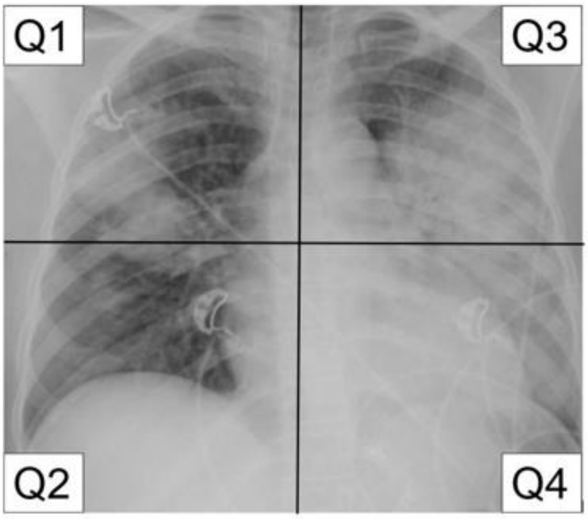


Density[b]

| Density Score | Density of alveolar opacities |
|---|---|
| 1 | Hazy |
| 2 | Moderate |
| 3 | Dense |

Final RALE Score[c]

| Right Lung | Left Quadrant |
|---|---|
| Upper Quadrant<br>Cons x Den = Q1 Score | Upper Quadrant<br>Cons x Den = Q3 Score |
| Lower Quadrant<br>Cons x Den = Q2 Score | Lower Quadrant<br>Cons x Den = Q4 Score |

**Total RALE = Q1 + Q2 + Q3 + Q4**

[a] Consolidation is scored for each quadrant
[b] Density is scored for each quadrant having a consolidation > 0
[c] If Quadrant consolidation Score is 0 then Quadrant score is 0

**eFigure 2. Study flowchart**

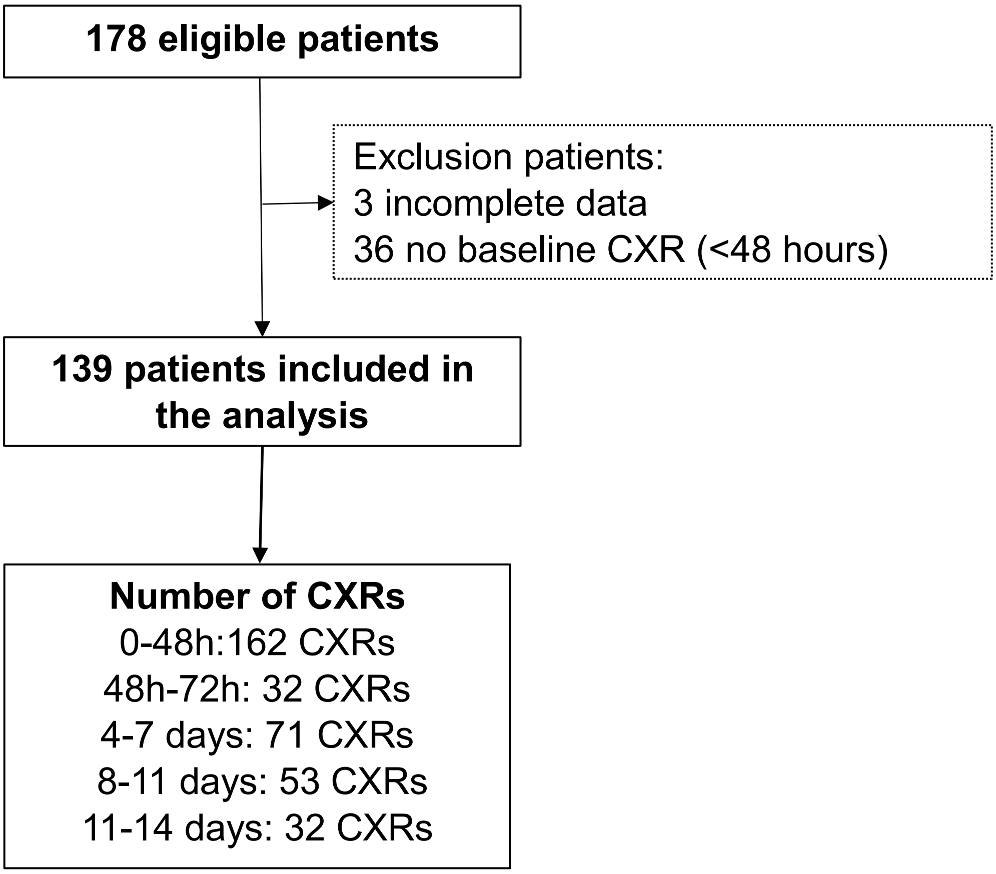

**eFigure 3. Dynamic compliance and RALE score values over time. Solid dots are means, solid lines are cubic spline regression fit and bandwidths are 95% bootstrapped confidence intervals.**

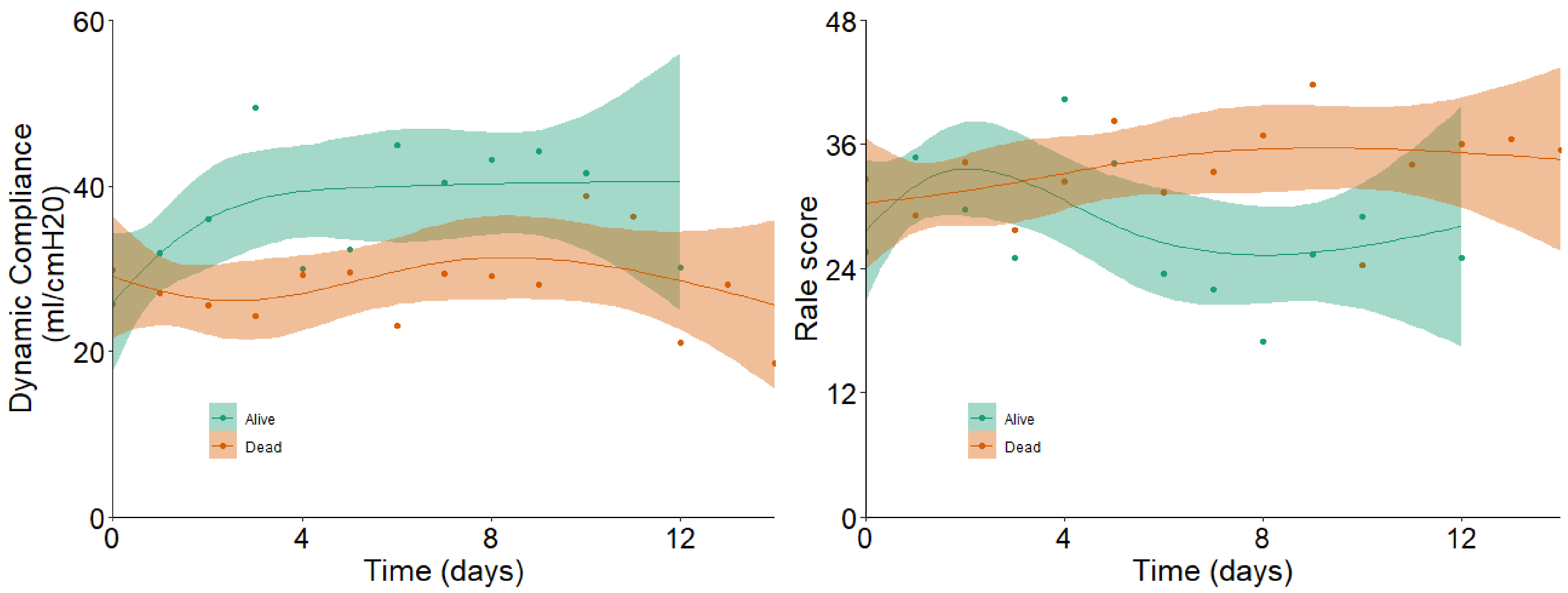

**eFigure 4. Bland Altman plots among the two independent scorers of study CXR**

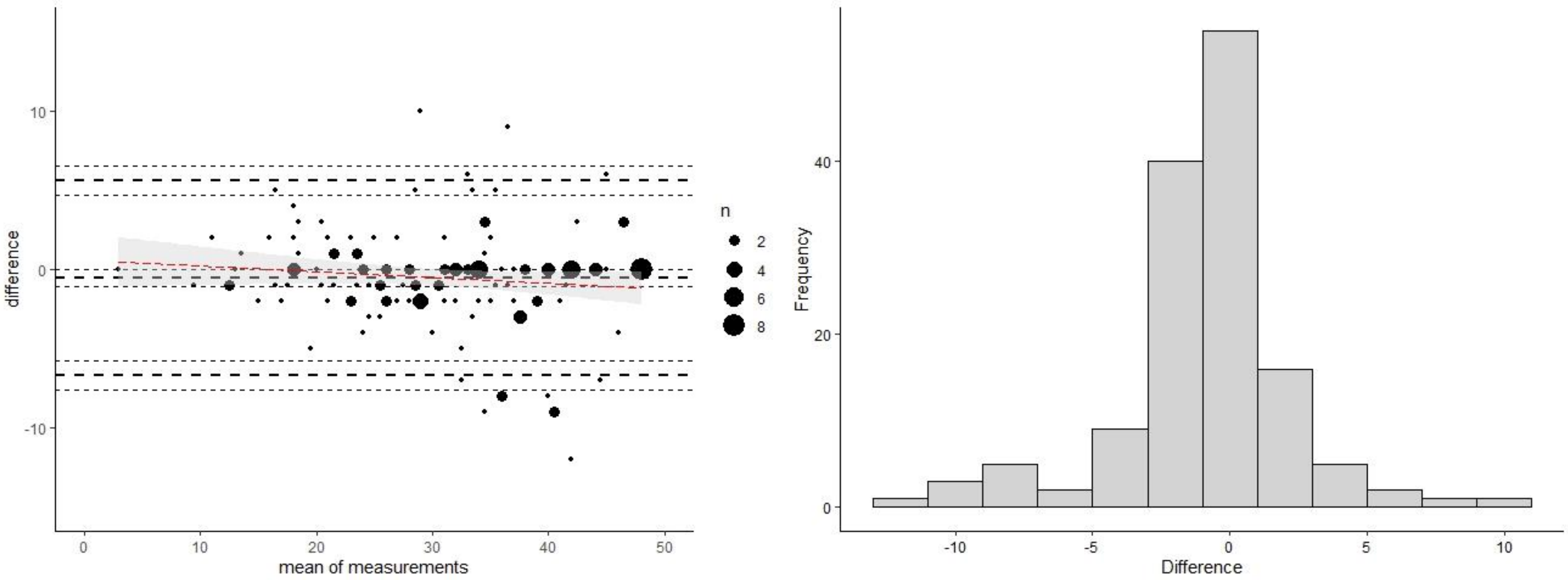


**Panel A:** Bland-Altman plot of agreement between RALE score readings. The bias is -0.5 [-1.0 – -0.1], lower limit of agreement -6 [-7 – -5], upper limit of agreement 5 [4 – 6]. Black dashed lines are the bias and limits of agreement with 95% confidence intervals. Red dashed line linear regression line assessing proportional bias. **Panel B:** Histogram of the of the observed differences between the RALE readings

**eFigure 5. Joint model prediction in a patient showing the change in prognosis based on RALE score as the longitudinal biomarker information since day of ICU admission.**

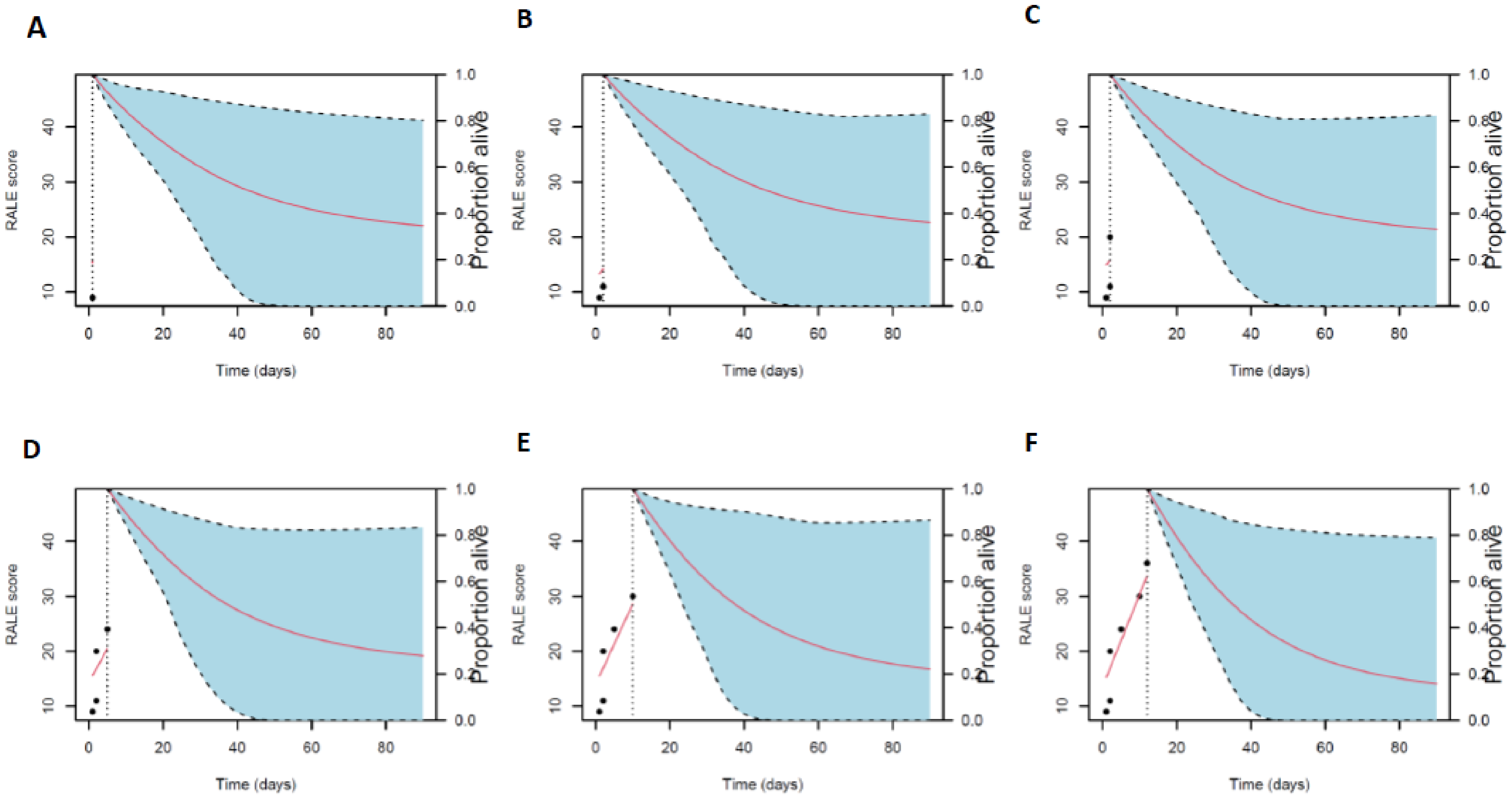


The longitudinal biomarker is showed on the left of the dashed line in each graph. Survival curves (red line) with 95% confidence interval bands (light blue) are depicted on the right side of the dashed line in every graph. **Panel A** shows the first RALE score available. At day 2 two chest X–rays were performed (**panel B** and **C**), and RALE score worsened. Three additional RALE score are recorded (**panel D–F**). As additional and higher RALE scores are introduced in the model, a substantial increase in the hazard of death is showed compared to baseline. This patient died 42 days after ICU admission.
